# Use of Segregation Indices, Townsend Index, and Air Toxics Data to Assess Lifetime Cancer Risk Disparities in Metropolitan Charleston, South Carolina, USA 

**DOI:** 10.3390/ijerph110505510

**Published:** 2014-05-21

**Authors:** LaShanta J. Rice, Chengsheng Jiang, Sacoby M. Wilson, Kristen Burwell-Naney, Ashok Samantapudi, Hongmei Zhang

**Affiliations:** 1 Department of Health Promotion, Education, and Behavior, University of South Carolina, 915 Greene Street, Columbia, SC 29208, USA; 2 Maryland Institute for Applied Environmental Health, University of Maryland, 255 Valley Drive, College Park, MD 20742, USA; E-Mails: cjiang89@umd.edu (C.J.); swilson2@umd.edu (S.M.W.); kburwell@umd.edu (K.B.-N.); 3 Department of Epidemiology and Biostatistics, University of South Carolina, 915 Greene Street, Columbia, SC 29208, USA; E-Mail: samantap@email.sc.edu; 4 Division of Epidemiology, Biostatistics, and Environmental Health, The University of Memphis, 3825 DeSoto Avenue, Memphis, TN 38152, USA; E-Mail: hzhang6@memphis.edu

**Keywords:** lifetime cancer risk, environmental justice, health disparities, NATA, air toxics, residential segregation

## Abstract

*Background*: Studies have demonstrated a relationship between segregation and level of education, occupational opportunities, and risk behaviors, yet a paucity of research has elucidated the association between racial residential segregation, socioeconomic deprivation, and lifetime cancer risk. *Objectives*: We examined estimated lifetime cancer risk from air toxics by racial composition, segregation, and deprivation in census tracts in Metropolitan Charleston. *Methods*: Segregation indices were used to measure the distribution of groups of people from different races within neighborhoods. The Townsend Index was used to measure economic deprivation in the study area. Poisson multivariate regressions were applied to assess the association of lifetime cancer risk with segregation indices and Townsend Index along with several sociodemographic measures. *Results*: Lifetime cancer risk from all pollution sources was 28 persons/million for half of the census tracts in Metropolitan Charleston. Isolation Index and Townsend Index both showed significant correlation with lifetime cancer risk from different sources. This significance still holds after adjusting for other sociodemographic measures in a Poisson regression, and these two indices have stronger effect on lifetime cancer risk compared to the effects of sociodemographic measures. *Conclusions*: We found that material deprivation, measured by the Townsend Index and segregation measured by the Isolation index, introduced high impact on lifetime cancer risk by air toxics at the census tract level.

## 1. Introduction

Racial and ethnic persons of color and disadvantaged populations in metropolitan areas are disproportionately exposed to unhealthy environmental conditions [1,2] and have increasingly higher death rates for most cancers [3]. Disparities in cancer have been attributed to complex structural inequalities including systemic differences in socioeconomic position and quality of neighborhood environments [3,4]. As the national health agenda moves toward eliminating health disparities [5], studies have focused on the role of environmental conditions including social and physical in causing and increasing health disparities [4,6,7,8,9,10]. Extensive literature has shown an association between residential segregation and various health outcomes including self-rated health [11], all-cause mortality [12], infant mortality [13,14,15,16,17], and air pollution exposure and lifetime cancer risk [18,19,20,21,22]. 

Macrosocial experiences over the life course create differential exposure to chemical, physical, and psyschosocial stressors for racial/ethnic and economically disadvantaged populations in areas known as “riskscapes” [23]. According to Wilson [2], “unhealthy community ecosystems” are a consequence of spatiotemporal disparities that operate through neighborhood segregation. There has been substantial research demonstrating that a lack of diversity in neighborhood racial composition produces racial disparities in educational opportunities [6,24], employment [6,25,26], and social and environmental conditions [6,27,28]. Williams and Collins [6] contend racial residential segregation is a root cause of racial health disparities given that it produces socioeconomic environments that lead to deleterious health outcomes. Correlations have been found between residential segregation and infant mortality [17], smoking during pregnancy [29], injection drug use [30], and breast cancer [27,31]. 

Russell *et al.* [31] observed a strong correlation between breast cancer mortality and survival for blacks *vs.* whites in metropolitan cities. Collins and Williams [32] identified an association between residential segregation and mortality across 107 major U.S. cities and found that racial isolation was associated with all-cause and cancer mortality for African Americans, even after adjusting for socioeconomic indicators. Jackson and colleagues [12] had similar findings in metropolitan areas with increasing levels of mortality. As the level of residential segregation increased, mortality risk and rates among African Americans increased. 

Morenoff *et al.* [9] concluded that black/white differences in hypertension were attenuated when statistical adjustments were made for neighborhood disadvantage. Do *et al.* [33] found that neighborhood context partially explained black/white differences in body mass index. Studies assessing associations between mortality in whites and residential segregation have been mixed. For example, some studies have found an inverse association between residential segregation and mortality for whites [13,34], while others have shown no association or a positive association [12,32]. 

Residential segregation operates through concentrated poverty and neighborhood disadvantage to affect health [6]. Robert and Ruel [35] documented weak associations between residential segregation and self-rated health when area socioeconomic characteristics were additionally considered in statistical models. However, Grady [36] found that neighborhood residential segregation was positively and significantly associated with low birthweight, after controlling for individual-level risk factors and neighborhood poverty. 

It is reasonable to assume that concentrated poverty is one of many pathways by which segregation impacts health but certainly not the only pathway. Residing in areas with high concentrations of poverty and economic disadvantage are positively associated with excess and all-cause mortality [6,27,37], cardiovascular disease [7], infant mortality [14,38], and poorer mental health [6,14,39,40]. 

Others have demonstrated a correlation between segregation, exposures to environmental risk factors (physical, social, economic), and health among disadvantaged populations in urban and rural areas [1,21,22,41,42,43,44]. Residential segregation exacerbates unhealthy conditions in the social and physical environment as well as limits the resources accessible to disadvantaged and low-income populations [6]. Urban macrosocial and political forces and the complex interplay of poor community investment, industrialization, and inequitable city planning and development have inhibited the growth and quality of neighborhood resources in communities of color [6,21,22,41,45]. 

These communities are differentially burdened by high levels of criteria air pollutants (CAPs) (e.g., carbon monoxide, particulate matter, sulfur dioxide, nitrogen oxides) released from vehicle exhaust due to heavy traffic loads on highways that bisect or border their neighborhoods and factories located in industrial corridors that are spatially concomitant with these neighborhoods [44,46,47]. Exposure to these pollutants can lead to cancer or exacerbate negative respiratory health outcomes (e.g., asthma) [21,22,44,46,47]. Morello-Frosch and Lopez [21] used regression analysis to examine the relationship between average levels of criteria air pollutants and Black-White residential segregation across metropolitan statistical areas (MSAs). After controlling for several socioeconomic status (SES) variables including percent (%) Black, poverty, and per capita income, Black-White segregation was correlated with increased levels of sulfur dioxide, PM_10_, and ozone across metropolitan regions.

For example, residential segregation in Metropolitan Charleston has steadily declined since 1990, resulting in a less segregated metropolis [34]. However, African-Americans primarily live in North Charleston [48], an area which hosts numerous Toxic Release Inventory (TRI) facilities [49] and greater than 50% of the block groups are occupied by persons of color, and socioeconomically disadvantaged persons including individuals living below the federal poverty line [50]. In contrast to other racial/ethnic groups and whites, African-Americans occupy areas that receive more public assistance [36], have higher levels of unemployment [36], and have more of its members living in close spatial proximity to others in their racial group [37]. There is limited information on the role that segregation, deprivation, and socioeconomic status have in driving lifetime cancer risk in Charleston, a historically segregated region undergoing rapid development and urbanization.

The purpose of this study was to assess the correlation between residential segregation, racial composition and socioeconomic factors and lifetime cancer risk due to air toxics at the census tract level in the Charleston Metropolitan Statistical Area (MSA). Building on previous research [18,19,20], we evaluated lifetime cancer risk disparities due to air toxics using segregation and deprivation indices in Metropolitan Charleston. 

## 2. Methods

### 2.1. Study Area

We focused our study on Charleston, South Carolina, a metropolitan region with known environmental justice issues [49,50,51,52,53]. Metropolitan Charleston is an area located on the coast of SC that is comprised of Berkeley, Charleston, and Dorchester counties. 

### 2.2. Census Data

Percent Black, unemployment, poverty, population with less than a high school education, households with more than two people living in a room, households without a car and owner occupied housing were calculated at the tract level using 2000 census data downloaded from the U.S. Census Bureau website. Topologically Integrated Geographic Encoding and Referencing (TIGER) geographic data of the Charleston MSA were also downloaded from the U.S. Census website. 

### 2.3. Segregation Index

Segregation indices were used in the analysis, which measure the distribution of groups of people from different races within neighborhoods. There are five dimensions of residential segregation, which will be examined separately: (1) evenness, (2) exposure, (3) concentration, (4) centralization, and (5) clustering [54,55]. The Dissimilarity Index [54,55] is a measure of evenness calculated by:

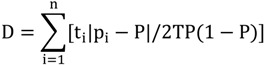
(1)
where t_i_ and p_i_ are the total population and proportion of non-white population in sub area i and T and P represent the population and non-white proportion within the total area. This index ranges from 0 to 1, indicating the proportion of non-white persons that are required to move to other residency areas to achieve evenness. The Isolation Index is a measure of exposure ranging from 0 to 1 that may be calculated by [56]:

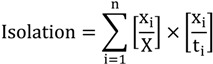
(2)
where x_i_ represents the non-white population in sub area i and x is the non-white population in all areas. The isolation equation illustrates the probability that two non-white individuals share the same unit. Delta is a measure of concentration defined by [56]:

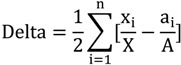
(3)
where a_i_ is the land area of sub area i and A represents the total land area. Delta not only considers the proportion relationship between non-white and majority group, but also the population density of non-whites. The Relative Cluster is an index of Clustering:

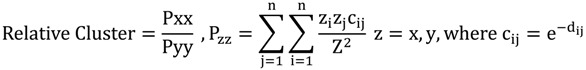
(4)
where x is the non-white group; y is the non-white group; z_i_ is the group population in sub area i; Z is the population in the whole area; and d_ij_ is the distance between sub area i and sub area j. The relative cluster compares the spatial proximity between non-white and majority groups.

The Townsend Index is a measure of economic deprivation within an area and is constructed by the percentage of households without a car, households with more than two people living in a room, house renter, and unemployment [57]:

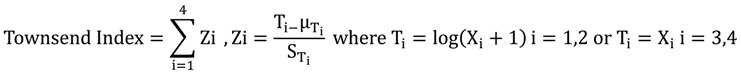
(5)
Where X_1_ and X_2_ are the percentage of unemployment and households with more than two people living in a room; and X_3_ and X_4_ are the percentage of households without a car and house renter. Furthermore, a high Townsend Index is indicative of high material deprivation. S_T_i__ is the standard deviation of T_i_.

### 2.4. NATA Risk

The EPA’s National-Scale Air Toxics Assessment (NATA) quantitatively evaluates 187 air toxics or hazardous air pollutants (HAPs) emission sources in the United States [58]. NATA estimates lifetime cancer risk by emission sources at the tract level. The NATA dataset includes five types of emission sources: (1) on-road (vehicles found on roads and highways); (2) non on-road (mobile sources not found on roads and highways, such as airplanes and farm machinery); (3) major (stationary facilities that emit or have the potential to emit 10 tons of any one toxic air pollutant or 25 tons of more than one toxic air pollutant per year); (4) area (sources that generally have lower emissions on an individual basis than “major sources” and are often too small or ubiquitous to be inventoried as individual sources) and (5) background sources. Lifetime cancer risk for all five emission sources were summed to calculate total lifetime cancer risk.

### 2.5. Statistical Analysis

NATA lifetime cancer risk data for each census tract were linked with census data by the Federal Information Processing Standard (FIPS) [59]. Due to collinearity among the variables, Kendall’s *Tau* was calculated to evaluate the association between NATA lifetime cancer risk and segregation index or demographic variables for each source of lifetime cancer risk.

Based on the findings from Kendall’s *Tau* tests, we examined the association of segregation and sociodemographic status with lifetime cancer risk by use of multivariate Poisson regression. Variables used in the calculation of segregation indices and those not showing high correlation with lifetime cancer risk were not included in the analyses. Furthermore, among the selected candidate variables, if there existed a strong correlation between some of the variables, we used one representative variable to avoid collinearity. The statistical analyses were conducted in R [60] and the significance level was set at 0.05.

## 3. Results

There are 117 census tracts in the Charleston MSA with the number of block groups within tracts ranging from 1 to 8. Segregation indices were calculated for each census tract using population data available at the block group level except for the Townsend Index which uses population data from the census tract itself. There were 12 tracts that had only one block group which made it impossible to calculate segregation indices for these tracts. Table 1 shows a summary of segregation indices, sociodemographic measures, and lifetime cancer risk. 

**Table 1:** Summary of segregation indices, townsend index, sociodemographic measures, and lifetime air toxics cancer risk.

Variable	Percentile			
	Mean	5th	50th	95th
**Segregation and Townsend Indices**				
Diversity	0.4	0.1	0.4	0.6
Isolation	0.4	0	0.4	0.9
Dissimilarit	0.2	0	0.2	0.6
Relative Cluster	0.1	−0.3	0	0.6
Delta	0.3	0	0.2	0.6
Townsend	1	−3.1	−0.2	8.8
**Sociodemographic Measures ^a^**				
% Black	35.5	0.7	26.3	90.4
% Unemployment	4.1	1	3	10.5
% Renter	37.7	9.6	32.5	82
% Crowded Room	3.6	0	3	9
% No Car	13.3	1.4	7.7	42.2
% Poverty	17.8	4.1	13.5	44.3
% Less than HS Education	20.9	3.1	20	41.7
**Lifetime cancer risk (persons/million) ^b^**				
All Source	29.1	22	28	42
Major	2.5	1	2	7.2
Area	2.1	1	2	4
On-Road	6.2	2	5	14
Non-Road	2	1	2	5
Background	16.3	15	16	18

Notes:^**a**^ Sociodemographic measures were used to derive Indices; **^b^** persons/million.

Mean % Black across all the tracts (35.5%) was slightly higher than the statewide mean (29.9%). According to the Diversity Index, the average probability that two people were randomly selected from different racial groups or ethnicities in a tract was 0.4. When the population was evenly distributed between different racial groups and ethnicities, the Diversity Index was expected to be 0.1, lower than our estimated probability of 0.4. Other indices also implied uneven distributions.

The Dissimilarity Index indicated that on average 20% of non-whites needed to move between block groups within tracts in order to attain an even racial/ethnic distribution. The Isolation Index indicates that the probability of two non-white individuals sharing the same block group was less than 0.4 for half of the tracts. Both Relative Cluster and Delta Indices illustrated that the non-white groups were more likely to live in the same community. The Townsend Index showed that at least half of the tracts had relative affluence (tracts with Townsend Index <0).

Median values for each sociodemographic factor were calculated. The median unemployment rate (3%) was lower than the national unemployment rate (5.8%) [60]. Furthermore, % renters (32.5%) was almost the same as the national rate (33.8%) [60]. The percentage of households without a car was also (7.7%) lower than the national rate (10.3%) (16). Moreover, % households with more than one occupant per room was 3% which was lower than the national rate (5.7%) [61]. The lifetime cancer risk of all sources exceeded 28 persons/million for half of the census tracts in the Charleston MSA, which means, on average, one in every 35,800 people have an increased likelihood of contracting cancer if they were exposed to 2005 air pollution emission levels in Charleston areas for 70 years. Lifetime estimated cancer risk using the NATA dataset was 50 persons/million for the country and 42 persons/million for the state, both numbers were higher than the estimated lifetime cancer risk for Charleston. Background sources were the largest single contributor (in most tracts) to lifetime cancer risk followed by mobile on-road sources.

**Figure 1:**
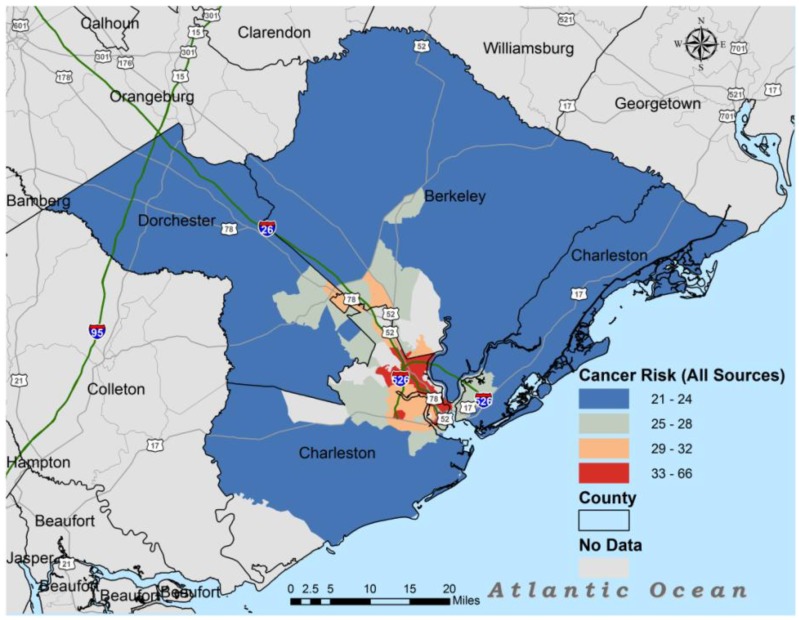
Lifetime cancer risk from all sources of air toxics for metropolitan Charleston.

**Figure 2:**
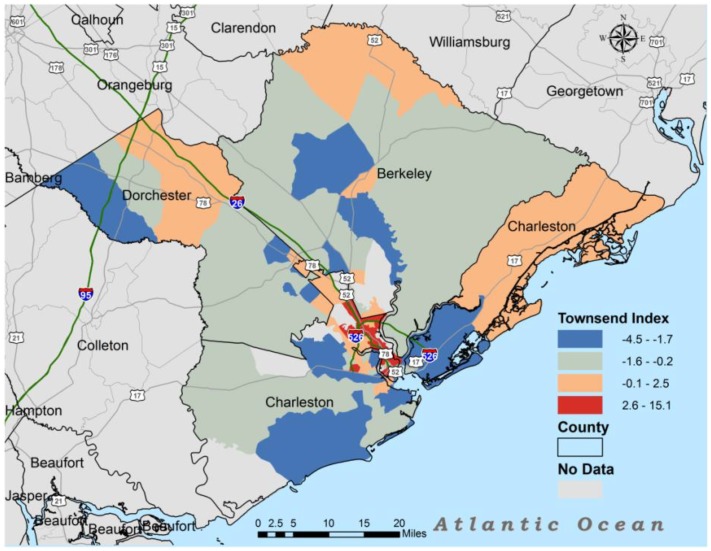
Townsend index for Charleston, South Carolina.

Figure 1 illustrates geospatial relationship between all source lifetime cancer risk in the Charleston MSA, while Figure 2 illustrates the geospatial relationship between the Townsend Index and lifetime cancer risk in the Charleston MSA.

The map in Figure 1 indicates that lifetime cancer risk was more concentrated around the intersection of route 26 and route 526 with some of the largest clusters found along Route 526. Not only do these areas have the highest lifetime cancer risk, but they also had the highest Townsend Index or greatest material deprivation (Figure 2). Most census tracts in Dorchester and Berkeley counties had NATA lifetime cancer risk less than 24 persons/million. The areas with Townsend Index less than −1.7 also had lower lifetime cancer risk except for areas closer to route 526 and route 26.

Table 2 displays the correlation between lifetime cancer risk and sociodemographic measures and segregation indices. The correlation between the Townsend Index and lifetime cancer risk from all sources was the highest (0.69) among all indices and sociodemographic measures. Moreover, the Townsend Index was most correlated with on-road and major sources when considering all indices and sociodemographic measures. Of the sociodemographic measures, % households without a car was most correlated (0.63) with on-road sources of lifetime cancer risk. The percent renter variable had the highest correlation with area and non-road risk (0.48 and 0.56, respectively) among all indices and sociodemographic measures. All correlations between lifetime cancer risk due to air toxics and Delta or Dissimilarity Index were not statistically significant.

We performed a Poisson multivariate regression to further evaluate the association of potential risk factors (segregation indices, Townsend index, and sociodemographic measures) with lifetime cancer risk from all sources of air toxics. We focused on all sources of lifetime cancer risk instead of individual sources of cancer risk. This is due to the information given in Table 2, which indicated that the potential risk factors are similar across different sources of lifetime cancer risk. Furthermore, due to the overlap of variables in the calculation of segregation indices and in the calculation of Townsend index, we fit different models with each model including one segregation index or Townsend index. As indicated in Table 2, since Isolation index and Townsend index showed significant correlation with most sources of lifetime cancer risk, we focused on these two indices. It was found that Isolation index, % unemployment, % renter, and Townsend index were significantly associated with lifetime cancer risk from all sources (Table 3). Specifically, the risk ratio was 1.25 (*p* < 0.01) for Isolation, 1.02 (*p* < 0.01) for % unemployment, 1.01 (*p* < 0.01) for % renter, and 1.03 (*p* < 0.01) for the Townsend index.

**Table 2:** Kendall’s *tau* between segregation indices, deprivation, sociodemographic measures and lifetime cancer risk from air toxics of different air pollution sources.

Segregation & Townsend Indices	All Source	Major	Area	On-road	Non-road	Background
Diversity	−0.04	−0.08	−0.01	−0.04	−0.1	0.03
Isolation	0.18 **	0.18 *	0.06	0.19 **	0.15 *	−0.06
Dissimilarity	−0.08	−0.08	−0.06	−0.06	0.05	0.05
Relative Cluster	−0.15 *	−0.11	−0.07	−0.17 **	−0.09	0.07
Delta	−0.02	0.03	−0.02	−0.01	0.03	0.06
Townsend	0.49 **	0.37 **	0.43 **	0.42 **	0.39 **	−0.41 **
**Sociodemographic Measures**						
% Black	0.21 **	0.16 *	0.09	0.2 **	0.14 *	−0.08
% Poverty	0.33 **	0.24 **	0.25 **	0.29 **	0.3 **	−0.27 **
% Less than HS	0.16 *	0.19 **	0.06	0.13	0.07	0.01
% Unemployment	0.27 **	0.27 **	0.19 **	0.23 **	0.22 **	−0.19 **
% Renter	0.55 **	0.38 **	0.52 **	0.49 **	0.52 **	−0.54 **
% Crowded Room	0.22 **	0.16 *	0.21 **	0.21 **	0.08	−0.09
% No Car	0.32 **	0.25 **	0.28 **	0.26 **	0.33 **	−0.22 **
% Urban Area	0.45 **	0.29 **	0.49 **	0.46 **	0.35 **	−0.36 **

Notes: *: *p*-value < 0.05; ** *p*-value < 0.01.

**Table 3:** Poisson multivariate regression of lifetime cancer risk from air toxics by isolation index and Townsend index.

Segregation Models	Coefficients (Exponential)
**Isolation Index Model ^+^**	
Isolation	1.25 **
% Poverty	0.99
% Unemployment	1.02 **
% Crowded Room	1.01
% Renter	1.01 **
**Townsend Index Model ^++^**	
Townsend	1.03 **
% Black	1.00
% Poverty	1.00

Notes: *: *p*-value < 0.05; ** *p*-value < 0.01; **^+^**: The variables included in this model were those showing a significant correlation (Kendall’s *Tau*) with lifetime cancer risk. Race was not included because isolation indices were calculated based on race. Furthermore, since the correlation between % Poverty and % No Car was 0.87, and was 0.83 between Isolation and % Less than HS, we excluded % No Car and % Less than HS; **^++^**: The variables included in this model were those showing a significant correlation (Kendall’s *Tau*) with lifetime cancer risk. Percent No Car, % Crowded Room, % Renter, and % Unemployment were not included because the Townsend Index was calculated based on these variables. Furthermore, since the correlation between % Black and % Less than HS was 0.85, we excluded % Less than HS from the model.

## 4. Discussion

This study assessed the correlation between sociodemographic characteristics, segregation indices, deprivation index, and lifetime cancer risk by sources of air toxics emissions in Metropolitan Charleston, SC. Consistent with other studies, most of the air toxics related cancer risk in Metropolitan Charleston was attributable to on-road sources (five persons/million) when excluding the risk from background sources. This region has a high number of vehicle miles traveled (VMT) per day (14,764,784) [62] and limited mass transit infrastructure, which corresponds to the high percentage of people who own a car (92.3%). These findings may also be due to traffic associated with the Port of Charleston, one of the top ten busiest ports in the U.S., and other port-related businesses [63]. With ongoing port expansion, the cumulative burden of these on-road sources may continue to increase lifetime cancer risk as diesel emissions and related pollutants (*i.e.*, particulate matter and black carbon) also increase.

Not only were on-road sources problematic, together with background, these sources accounted for roughly 75% of the lifetime cancer risk due to air toxics in Metropolitan Charleston. While background sources are always present and are comprised of natural sources of air toxics, chemicals persisting in the environment from previous year’s emissions, and other air toxics transported from distant sources can still significantly increase lifetime cancer risk [58]. Major, area, and non-road sources were all equal contributors to lifetime cancer risk in Metropolitan Charleston; however, non-road sources most correlated with sociodemographic measures and segregation indices of interest.

This finding was unexpected due to the high concentration of air toxics released from major sources in the area. For example, a recent study by Wilson *et al.* [49] found that there were 63 toxic release inventory (TRI) facilities in Metropolitan Charleston releasing approximately 17 million pounds of contaminants. While the TRI facilities in Metropolitan Charleston only accounted for 12.4% of the facilities in the entire state (510), they still contributed to 26% of SC’s total releases [49]. The high toxic releases in Metropolitan Charleston may explain why major sources were a major contributor to lifetime cancer risk (Table 1) despite the lower correlations found among the sociodemographic measures and segregation indices (Table 2).

Lopez [64] evaluated Black-White disparities in air toxics exposure across large metropolises (*i.e.*, >1 million people). Using the Dissimilarity Index, Lopez [64] observed an increase in residential segregation by air toxics exposure and proposed the usefulness of segregation indices in approximating racial disparities in air pollution. Contrary to the aforementioned findings, we did not observe a correlation between air toxics and Dissimilarity Index. We did find that the Townsend Index was most correlated with all sources of lifetime cancer risk (Table 2), which implies that economic deprivation may be more associated with lifetime cancer risk than racial composition. Our findings also indicated that Townsend Index and Isolation Index show higher impact compared to other sociodemographic measures such as % black. The SES indicators that comprise the Townsend Index (% households without a car, % households with more than two people living in a room, % renters, and % unemployment) demonstrate the vulnerability of the population in Metropolitan Charleston to lifetime cancer risk and their ability to cope with related poor health outcomes.

Further adding to the burden of lifetime cancer risk due to air toxics in Metropolitan Charleston is that cancer risk differentially affects non-white and economically disadvantaged population. For example, the Charleston MSA is approximately 90% Black in some census tracts and has a higher unemployment rate (10.5%) than the state (8.1%). When considering segregation in a population most impacted by air toxics, the Isolation and Townsend Indices were significantly associated with lifetime cancer risk after controlling for other effects (Table 3). For example, a one-unit increase in the Isolation Index increased lifetime cancer risk by 25% after controlling for % poverty, % unemployment, % crowded room, and % renter. Only % unemployment and % renter was statistically significant, which demonstrates that the Isolation Index was a better predictor of lifetime cancer risk compared to using the single measures previously mentioned. Moreover, a one-unit increase in the Townsend index increased lifetime cancer risk by 3% while % black and % poverty were not statistically significant when included in the model. Similar to the Isolation Index, the Townsend index was a better predictor of lifetime cancer risk instead of individual factors that are used to construct the index.

Studies have documented disparities in lifetime cancer risk and other adverse health conditions among non-white and low-income populations [20,47]. In addition to disparities in lifetime cancer risk, these vulnerable populations may be less likely to have the health promoting infrastructure to cope with health effects associated with exposure to air toxics or other environmental stressors [43]. According to a study by Acevedo-Garcia *et al.*, the hypothesized pathway between residential segregation and health may be indirectly explained by the quality of neighborhood environment attributes and SES as it relates to limited employment and educational opportunities [65]. This particular phenomenon has been further documented as the racial income inequality thesis, which states that racial differences in exposure to various environmental hazards are linked to inequities in socioeconomic resources [66]. Specifically, a neighborhood that is overburdened by environmental hazards is more likely to have lower property values and may be more appealing to low-income families compared to more affluent populations [66]. Since community resources (*i.e.*, property taxes) dictate the quality of neighborhood schools, racial residential segregation may facilitate an environment that is not conducive to students seeking higher education [6]. As a result, high-paying employment opportunities may be limited and the cycle continues where predominately non-white and low-income populations reside near environmental hazards that increase cancer risk and other negative health outcomes [20,47].

While the cycle that facilitates racial residential segregation still exists, there are ways that we can reduce its impact across Charleston, SC. For example, more mixed-income housing communities can be created in Charleston in order to decrease the probability of any one racial or socioeconomic group being overrepresented in a particular area. This would allow non-white and low-income populations to have better access to other housing options, improved neighborhood environments with fewer environmental hazards, better schools, higher-paying jobs, and ultimately decrease health disparities related to air pollution and other exposures. In addition, improvements in Charleston’s public transportation system could help reduce cancer risk disparities attributable to on-road sources of air toxics. As previously mentioned, Charleston has a high number of vehicle miles traveled with most person’s owning a car (92.3%). If we could build upon the current mass transit infrastructure so that people have access to public transportation, then we may begin to see reductions in air pollution and hence decreases in cancer risk associated with on-road sources.

## 5. Conclusions

The lifetime cancer risk disparities we observed were primarily attributable to on-road sources of pollution. Our findings suggest high isolation influences lifetime cancer risk and the Townsend index should be considered as a strong measure of lifetime cancer risk disparities from air toxics due to its high correlation and statistical significance with lifetime cancer risk sources as well as sociodemographic measures. Our study emphasized the importance of place in understanding lifetime cancer risk disparities and the need to focus future efforts on assessing socioeconomic and neighborhood level drivers of environmental health risk including air toxics-related cancer risk. Furthermore, this investigation provides a preliminary assessment of the shared socioeconomic and neighborhood experiences that may be contributing to cancer and other health disparities as well as the need for better desegregation policies and efforts to implement equitable planning, zoning, and community development policies to improve access to environmental and health resources for underserved and health disparity populations.
